# Effect of Hip Muscle Strengthening Exercises on Pain and Disability in Patients with Non-Specific Low Back Pain—A Systematic Review

**DOI:** 10.3390/sports11090167

**Published:** 2023-09-01

**Authors:** Gema Santamaría, Irene Rodríguez, Vicente Rodríguez-Pérez, Raúl Cobreros-Mielgo, Eva Lantarón-Caeiro, Marina Seco-Casares, Diego Fernández-Lázaro

**Affiliations:** 1 Department of Anatomy and Radiology, Faculty of Health Sciences, Campus of Soria, University of Valladolid, 42003 Soria, Spain; gema.santamaria@uva.es; 2 Vitalia Home Cervera, 34840 Cervera de Pisuerga, Spain; irene20899@gmail.com; 3 Faculty of Nursing and Physiotherapy, Department of Nursing and Physiotherapy, Universidad de Salamanca, C/ Donantes de Sangre s/n, 37007 Salamanca, Spain; vicente.rodriguez@usal.es; 4 Institute of Biomedicine (IBIOMED), Physiotherapy Department, University of Leon, Campus de Vegazana, 24071 Leon, Spain; rcobm@unileon.es; 5 Physiotherapy Group FS1, Department of Functional Biology and Health Sciences, Faculty of Physical Therapy, University of Vigo, 36005 Pontevedra, Spain; evalantaron@uvigo.es; 6 Nursing Department, León University Assistance Complex (CAULE), Hospital of León, 24008 Leon, Spain; 7 Department of Cellular Biology, Genetic, Histology and Pharmacology, Faculty of Health Sciences, University of Valladolid, Campus de Soria, 42003 Soria, Spain; 8 Neurobiology Research Group, Faculty of Medicine, University of Valladolid, 47005 Valladolid, Spain

**Keywords:** low back pain, hip, strengthening, treatment, pain, disability

## Abstract

Low back pain (LBP) is a health problem that affects 70–80% of the population in Western countries. Because of the biomechanical relationship between the lumbar region and the hip, it is thought that strengthening the muscles of this joint could improve the symptoms of people with LBP. The objective of this study is to evaluate the current evidence on the efficacy of hip strengthening exercises to reduce pain and disability in people with LBP. Clinical trials were collected from the PubMed, PEDro, and Scopus databases published up to September 2022. Based on the Preferred Reporting Items for Systematic Review and Meta-Analysis (PRISMA) guidelines and using CASP and PEDro tools for methodological quality assessment, we selected studies that included hip strengthening exercises as part of LBP treatment and measured pain and/or disability parameters. Among the 966 records identified in the search, a total of 7 studies met the established selection criteria. Overall, participants who performed hip strengthening exercises had significantly improved in pain and disability. The methodological quality of the included studies was assessed as “good”. In conclusion, the addition of hip muscle strengthening exercises iterating interacted with LBP, effectively improving pain and disability.

## 1. Introduction

Low back pain (LBP) is an increasingly common condition worldwide, but in practical terms it is estimated that 70–80% of the population from countries of the Western world will suffer LBP at some point in their lives, especially affecting women over 40 years old [1,2]. This makes LBP the second most frequent chronic skeletal muscle pathology after osteoarthritis [1]. The history of LBP is the most consistent with and the main cause of general mobility restriction, long-term disability, and decreased quality of life (QoL); this is because the pain does not specifically limit the movement of a joint, but rather the pain is the cause of limiting general mobility in the daily life of patients [1,3]. It is noteworthy that the overall healthcare cost analysis of LBP is estimated in the range of USD 100 billion per year in the United States of America, including direct tangible costs, indirect costs of labor, productivity slowdowns, and monetary compensations [4]. Although most episodes of LBP usually resolve spontaneously a few days after their onset, a substantial proportion of patients, approximately 5–10% of the population, will develop chronic (duration > 3 months) or recurrent pain [1,5]. In 85% of cases, LBP is considered as non-specific pain, which means that no structural change, no inflammation, and no specific disease can be found as its cause [6]. This type of LBP is often associated with psychosocial factors and abnormal pain-coping behaviors [1].

One of the main problems of low back pain is the variety of treatments which occasionally are not harmonized with what has been reported by scientific evidence, worsening the results, chronifying pain, and substantially increasing healthcare costs [7,8]. During the acute phase (first 2–3 days), low back pain must be treated with rest and drugs (anti-inflammatory and/or analgesics), but if the pain persists, maintaining rest favors chronification [1]. For this reason, therapeutic exercise could currently be established as the most useful intervention in the treatment of LBP [9]. Therapeutic exercise in LBP would relieve pain, improve functionality, and reduce the risk of recurrence [9]. It is necessary to consider the entire spectrum of different exercise therapies, including motor control exercises, balance, aerobic training, stretching, and muscle strengthening [9].

The lumbar spine is biomechanically connected to the pelvic and hip joint, making it difficult to determine the provenance of symptoms in clinical practice [10]. The normal range of movement (ROM) of the hip is often altered in patients with LBP, making it impossible to correctly transmit the load from the lower limb (LL) to the trunk [11,12]. This is usually due to shortening of the flexor muscles, which limits coxofemoral extension and therefore increases lumbar extension, leading to lordosis [11,12]. On the other hand, it is common to find strength deficiency of the hip abductor and extensor muscles in patients suffering from LBP [12,13,14]. This shortage is usually compensated by over use of the hamstring muscles, which can lead to their curtailment and increased compensatory movements of the spine [12]. For this reason, studies and guidelines have recently begun to include hip strengthening exercises as part of the treatment of low back pain. [14]. In this sense, de Jesus et al. [14] has described that the inclusion of specific hip strengthening exercises in conventional rehabilitation therapy for low back pain attenuates painful symptoms and disability. However, this review did not include quality of life, hip and lumbar muscle strength, and balance. For this reason, it could be considered necessary to carry out more up-to-date studies that include these parameters due to the high prevalence of non-specific low back pain. Therefore, the objective of this systematic review is to analyze the existing scientific evidence on the effectiveness of hip muscle strengthening (HMS) and the reduction in related pain and disability in people with low back pain through analyzing quality of life, hip and lumbar muscle strength, and balance.

## 2. Materials and Methods

### 2.1. Search Strategy

For article selection, a structured search was carried out using the electronic databases Medline (PubMed), “Physiotherapy Evidence Database” (PEDro), and Scopus until September 2022. The PICOs model was used according to the standard methods proposed by the Preferred Reporting Items for Systematic Reviews and Meta-Analyses (PRISMA) [15] guidelines as follows: P (population), adults over 18 years who suffer from LBP; I (intervention), hip strengthening exercises (HSEs); C (comparison), control/placebo group, without treatment, or with any other treatment technique that does not involve the hip; O (outcomes), effects on pain (Visual Analogue Scale (VAS) and Numeric Pain Rating Scale (NPRS)), level of disability (Roland–Morris Disability Questionnaire (RMDQ), Oswestry Disability Index (ODI), and patient-specific functional scale (PSFS)), strength and resistance of the lumbar and hip muscles (dynamometry), flexibility of the hip muscles, gait analysis, balance, and QoL S (study design), clinical trial or randomized clinical trial.

The search strategy contained a combination of “Medical Subject Headings” (MeSH) and free words for related key concepts including the following: (“low back pain” OR “Mechanical” OR “ache, low back” OR “aches, low back” OR “Chronic low back pain” OR “lumbago” OR “non-specific low back pain” OR “lumbar instability”) AND (“hip” OR “hip mobility” OR “hip flexibility” OR “hip extensibility” OR “hip strength” OR “hip strengthening” OR “hip treatment” OR “hip intervention” OR “hip exercises” OR “gluteus” OR “aquatic exercise”) AND (“randomized controlled trial” OR “clinical trial” OR “trial”). Two authors (G.S. and D.F.-L.) independently performed the search for published studies and a third reviewer (I.R.) resolved disagreements about records. All the studies obtained in the 3 databases were compared in order to limit the search as much as possible and avoid repetition of studies. A review of all existing meta-analyses and systematic reviews was carried out to avoid losing studies due to lack of data search terms. Full-text articles were retrieved and checked for compliance with inclusion and exclusion criteria.

### 2.2. Selection Criteria

a. Inclusion Criteria

For inclusion in this review, studies had to (1) access the adult population with LBP; (2) treat using HSEs as the main intervention or in conjunction with other interventions; (3) compare with the group without intervention, with placebo treatment, or receiving another type of treatment non-related to hip; (4) include studies reporting primary or secondary outcomes related to pain (VAS and NPRS) and level of disability (RMDQ, ODI, and PSFS); (5) be clinical trials or randomized clinical trials with a score of 6 or more on the Critical Appraisal Skills Programme (CASP) questionnaire and the PEDro scale; (6) be published in Spanish or English.

b. Exclusion criteria

Studies were excluded from the review if they (1) included a population under 18 years or no age was specified; (2) reported that subjects had specific LBP (tumors, hernias, ankylosing spondylitis, fractures, etc.); (3) were reviews, meta-analyses, editorials, or non-original studies; (4) did not have an affirmative answer to the first three questions of the CASP questionnaire; (5) reported insufficient data or did not provide access to the full text.

### 2.3. Extraction and Synthesis of Data

A checklist for data extraction was developed from each study included in the review. The following study details were extracted: first author’s last name; year of publication; country where the study was conducted; design; sample size; sex; age; height; weight; intervention in the control group (CG) and intervention group (IG), focusing especially on the HSE protocol (exercises, volume and intensity, frequency, session time, study duration, and supervision); measurement scales used; and final results. Two researchers (G.S and I.R) carried out the data extraction process with the help of a spreadsheet. In the case of disagreements, a third author (D.F.-L.) participated in the process.

### 2.4. Assessment of Methodological Quality

The methodological evaluation of the selected trials was carried out using the PEDro [16] and CASP [17] scales, with the aim of excluding studies with deficient methodologies.

## 3. Results

### 3.1. Selection of Studies

**Figure 1:**
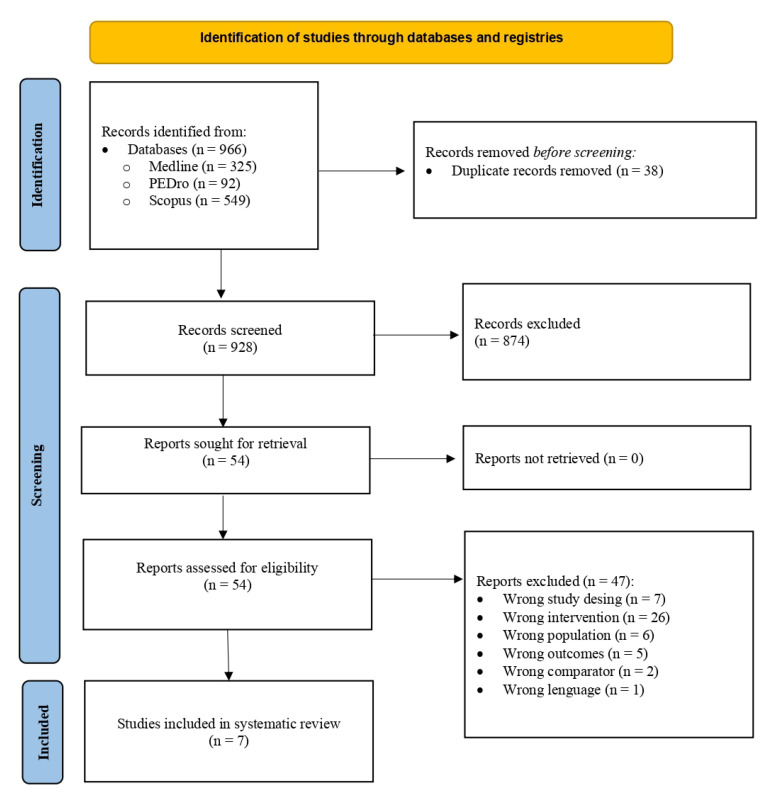
Flow chart of study selection for the literature review (PRISMA).

The search identified 966 potentially relevant studies in the three databases, 325 from PubMed, 92 from PEDro, and 549 from Scopus. After eliminating duplicates and reading the titles, 912 articles were discarded. In a second phase, 47 were eliminated due to the following: being non-clinical trials (n = 7), not training hip muscles (n = 26), not having a representative population (n = 6), not measuring pain and/or disability (n = 5), not presenting a CG (n = 2), and not being written in Spanish or English (n = 1). Additionally, the bibliographies of the included articles and some of the discarded ones were reviewed to search for new studies but none of interest were found. Therefore, after this search, seven articles were obtained that are included in this systematic review (Figure 1).

### 3.2. Assessment of Methodological Quality

All the included studies met the minimum methodological quality requirements with a score equal to or greater than 6, that is, “good”. The scores varied between 7 and 10 points on the CASP scale (Table 1) and between 6 and 9 on the PEDro scale (Table 2).

Due to the type of intervention that is intended to be studied, none of the studies met the requirement of complete blinding, since the therapists will always know the treatment they are performing and, therefore, to which group each patient belongs. Only the study carried out by Kim and Yim [23] kept the participants and evaluators blinded, while in those of Jeong et al. [21] and Lee et al. [24] nothing is specified about blinding.

### 3.3. Characteristics of Participants and Interventions

The characteristics of the participants are shown in Table 3. The total number of volunteers was 517, 230 women and 200 men aged between 18 and 77 years. Five of the studies used a sample composed of both men and women [18,19,20,22,23], one of them did not specify the number of participants of each sex [24], and the remaining study included only women [21].

All the studies compare HMS exercise programs (IG) and those directed by chest or of general nature (CG). Only the trial by Cai et al. [19] applied HMS as the sole treatment of the IG. In the remaining six studies, the IG, in addition to the HMS exercise program, received the same treatment as the CG, consisting of manual therapy of the lower back and/or hip joint, strengthening and resistance exercises of the trunk muscles, peripheral nervous system (PNS) mobilizations, aerobic exercise, fitness, education, motor control exercises, and lumbar stabilization (Table 3).

Table 4 shows the specific characteristics of the HMS protocols used in the IG. The study carried out by Cai et al. [19] was the only one to include strengthening exercises for the muscles involved in the knee joint. For their part, Kim and Yim [23] divided the IG into two subgroups: one of them performed HMS exercises and the other static stretching of the hamstrings, iliopsoas, piriformis, and tensor fasciae latae. All the studies focused on the work of the abductor and extensor muscles [18,19,20,21,22,23], and only Lee et al. [24] added the adductor muscles and the rotators. The duration of the intervention was similar in all studies, with a minimum of 5 weeks [20] and a maximum of 8 weeks [19].

### 3.4. Evaluation of the Results

a. Pain

Six [18,19,20,22,23,24] of the seven studies included in the review measured changes in pain with a total of 230 CG and 222 IG participants. Four studies [20,22,23,24] used the VAS scale to measure pain and the remaining two [18,19] used the NPRS scale. In all of them, an improvement or even pain relief was observed after the intervention in the IG, but the difference between groups was only statistically significant (*p* < 0.05) in three [18,19,23], one found non-significant improvements (*p* > 0.05) [24], and two did not find any difference [20,22] (Table 3).

b. Disability level

The level of disability was taken into account by the seven studies [18,19,20,21,22,23,24], with 250 participants belonging to the CG and 267 to the IG. Of these seven studies, one [20] used the Roland–Morris questionnaire, four [18,21,22,24] used the original or modified ODI, Kim and Yim [23] used both, and the last one [19] used the PSFS scale. As for pain, all the trials obtained improvements in the IG; however, this improvement was significantly greater (*p* < 0.05) in the IG compared to the CG only in four [18,19,21,23] (Table 3).

c. Other parameters evaluated

As can be seen in Table 3, three of the seven studies analyzed the strength of the hip muscles through dynamometry [19,20,22]. However, even though all the studies found improvements in the IG in comparison to the baseline, none were able to demonstrate statistically significant changes (*p* > 0.05) compared to the CG. In parallel, Cai et al. [19] and Jeong et al. [21] studied lumbar resistance and strength, respectively, and both found two statistically significant improvements (*p* < 0.05) in the IG compared to the CG. Kim and Yim [23] demonstrated statistically significant increases (*p* < 0.05) in the two IG versus the CG in QoL, studied with the SF-36 scale.

## 4. Discussion

All seven studies that met the inclusion/exclusion criteria found that HMS treatment for LBP may be effective in reducing both pain and disability in contrast to other non-hip interventions. Additionally, no unwanted effects have been reported in any of the subjects included in the studies, postulating that HMS as a safe and effective therapeutic exercise option.

According to the World Association for the Study of Pain (IASP), pain is considered “an unpleasant sensory and emotional experience associated with actual or potential tissue damage, or described in terms of such damage” [25]. Pain is subjective and should not always be eliminated, as it acts as a defense mechanism, protecting the body from dangerous situations. However, sometimes pain becomes a source of suffering, especially when appearing in the absence of tissue damage, frequently due to psychological disorders [25,26]. For its part, disability related to LBP makes it difficult to perform activities of daily living (ADL) and work tasks [27]. Additionally, LBP can lead the individual to social isolation and to avoid daily activities, reducing their self-efficacy and increasing the chances of developing depressive symptoms and disability [28]. In his way, aerobic exercise programs can produce a substantial improvement in mood and reduce depression in chronically ill patients [29]. Five of the studies [18,19,21,23,24] found statistically significant improvements (*p* < 0.05) in both pain and disability compared to the CG and seven [18,19,20,21,22,23,24] in the IG compared to baseline. This incongruity observed in results is likely due to the intensity, frequency, and duration of the interventions. The number of weekly sessions carried out in the study by Fukuda et al. [20] was two, and Kendall et al. [22] indicate that only one face-to-face session was given weekly, without specifying the number of weekly sessions at home, the duration of the sessions, or details about the volume and intensity (number of exercises, series, repetitions, rest times, etc.) of the same. This differs with the number of weekly sessions carried out in the interventions of the studies that obtained improvements in comparation to the CG, ranging from three to seven [18,19,21,23,24]. Additionally, the duration of treatment was shorter. Fukuda et al. [21] conducted a 5-week intervention and Kendall et al. [22] a 6-week intervention, while the duration in the rest of the studies was 6 to 8 weeks [18,19,21,23,24].

**Figure 2:**
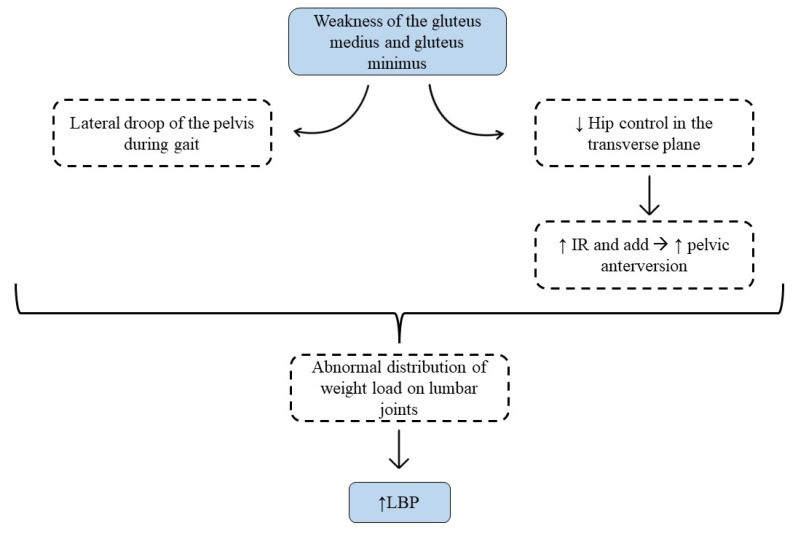
Description of the mechanism for how gluteal weakness increases low back pain.

Although the mechanism by which HMS exercises reduce pain and disability levels is not well understood, it may be due to the increase in pelvic stability provided by strengthening of the gluteal muscles [14,30]. The gluteus medius and minimus are responsible for controlling the position and stability of both the hip and the pelvis, so their weakness can lead to biomechanical changes in the coxolumbopelvic complex, contributing to LBP [30]. Mainly it will lead to the lateral descent of the pelvis while walking, which is known as the Trendelenburg sign. This will cause an abnormal distribution of weight load on the intervertebral discs and lumbar joints [30]. Additionally, gluteal weakness can lead to less control of the hip in the transverse plane, increasing internal rotation and adduction of the femur, which leads to an increase in pelvic anteversion and again results in abnormal load distribution at the lumbar level [11] (Figure 2). However, for the correct functioning of the coxolumbopelvic complex, not only an adequate level of force is necessary, but it is also important that the hip and lumbar ROM are maintained [11]. Techniques to increase ROM such as manual therapy or stretching could be useful adjuncts to improve pain and disability in patients with LBP, as shown in three studies included in this review [18,23,24]. In this sense, Kim and Yim [23] divided the IG into two: one performed static stretching of the hip muscles and the other HMS, and both found statistically significant improvements (*p* < 0.05) with respect to the CG and the baseline, with no differences between the two IGs in count pain and disability. However, they found statistically significant increases (*p* < 0.05) compared to the CG in QoL and lumbar stability in the IG who performed stretching. These increases were not observed in the IG with HMS exercises, demonstrating the importance of preserving the lumbar and pelvic–femoral ROM in the treatment of LBP.

The results described in the seven studies included in this review are consistent with those reported by Tataryn et al. [31], who obtained improvements in pain and disability both in the IG and in the CG; however, these were higher in the IG. These authors carried out a systematic review with a meta-analysis in which they intended to compare the effectiveness of exercises to strengthen the posterior chain of the LL and general exercise programs. This could be explained because LBP is associated with alterations in muscle activation patterns, strength, endurance, and flexibility and poor physical condition. This is confounded in part by conscious or unconscious avoidance behaviors for fear of worsening the problem [32,33]. This inactivity usually means a decrease in lumbopelvic stability and a greater load on the lumbar joints [33]. Therefore, exercise, whether for motor control, strength, flexibility, or resistance, will be effective in the treatment of LBP by improving pain and function. In particular, strength-training programs are considered essential to increase lumbopelvic stability [32,33].

### 4.1. Potential Applications

Considering the different protocols and results obtained in this study, we developed a therapeutic exercise intervention protocol with the aim of guiding clinical practice (Table 5). The training sessions should be structured in three parts, first with a warm-up with joint mobility exercises and muscle activation. The main part is where the HMS exercises are carried out, such as squats, Monster Walk, gluteal kick, lateral clam, gluteal bridge, and finally returning through relaxation exercises towards a calmer state. Importantly, static stretching and manual therapy of the coxofemoral joint are crucial through these sessions. Therefore, we would fulfill the key points of LBP treatment that we have developed throughout the discussion, specifically the HMS of the gluteus and the maintenance of the hip ROM. At the same time, it would also be interesting to include exercises to strengthen lumbar muscles and motor control, in addition to manual therapy techniques specifically targeting the lumbar spine. In relation to the workload, two to three series of 8–12 repetitions per exercise should be performed with a minute of rest between series and an intensity of 75–80% of one maximum repetition (RM). The duration of the sessions is approximately 60 min and may be conducted in 3–4 weekly sessions.

### 4.2. Limitations and Strengths

The authors of this review acknowledge some limitations. First, a limited number of manuscripts met the inclusion/exclusion criteria. Given the type of intervention studied it was impossible for the therapists to remain blinded, and only the trial by Kim and Yim [23] achieved blinding of patients and assessors. However, in order to minimize the risk of bias, the PRISMA method [15] was followed and the search was carried out in three databases. The CASP [17] and PEDro [16] tools were used for quality assessment and methodology and to ensure that the selected studies met the minimum quality criteria. Second, it was not possible for us to perform a meta-analysis due to the heterogeneity of samples and interventions, as well as the different scales and tests used to evaluate each parameter. Finally, the results should be interpreted considering the great heterogeneity in the studies, such as in terms of interventions (type of exercises, intensity, volume, frequency, duration of the sessions, and duration of the intervention) and the characteristics of the samples (age, sex, and level of physical activity). We did not register those protocols in any database/registry before conducting or publication.

## 5. Conclusions

The results presented in this systematic review showed that the inclusion of HMS exercises in an exercise protocol that involves the whole musculature or specifically targets the lower back provides significant improvements in the reduction in pain and disability in patients with LBP, without causing injury.

## Figures and Tables

**Table 1:** Results of methodological quality assessment of included studies—Critical Appraisal Skills Programme (CASP).

Study	Item											Total
	1	2	3	4	5	6	7	8	9	10	11	
Bade M et al. 2016 [18]	Yes	Yes	Yes	No	Yes	Yes	No	*p* < 0.05	Yes	Yes	Yes	9
Cai C et al. 2017 [19]	Yes	Yes	Yes	No	Yes	Yes	Yes	95% CI *p* < 0.01	No	Yes	Yes	9
Fukuda TY et al. 2021 [20]	Yes	Yes	Yes	No	Yes	Yes	No	95% CI	Yes	Yes	No	8
Jeong UC et al. 2015 [21]	Yes	Yes	Yes	Cannot tell	Yes	Yes	Yes	*p* < 0.01	No	Yes	Yes	9
Kendal KD et al. 2014 [22]	Yes	Yes	Yes	No	Yes	Yes	No	95% CI	Yes	Yes	No	8
Kim B and Yim 2020 [23]	Yes	Yes	Yes	No	Yes	Yes	Yes	*p* < 0.05	Yes	Yes	Yes	10
Lee SW et al. 2014 [24]	Yes	Yes	Yes	Cannot tell	Yes	Yes	Yes	*p* < 0.01	Yes	Yes	Yes	10

CASP questionnaire items → 1: clearly defined question; 2: random assignment; 3: patients considered until the end; 4: blinding; 5: similar groups at baseline; 6: equally treated groups; 7: longer treatment effect; 8: accuracy of effect; 9: applicability to your setting or local population; 10: all outcomes considered; 11: benefits justify risk and cost. Abbreviations → CI: confidence interval.

**Table 2:** Results of methodological quality assessment of included studies—Physiotherapy Evidence Database (PEDro).

Study	Item											Total
	1	2	3	4	5	6	7	8	9	10	11	
Bade M et al. 2016 [18]	Yes	Yes	No	Yes	No	No	No	Yes	Yes	Yes	Yes	7
Cai C et al. 2017 [19]	Yes	Yes	Yes	Yes	No	No	No	Yes	No	Yes	Yes	7
Fukuda TY et al. 2021 [20]	Yes	Yes	Yes	Yes	No	No	No	Yes	Yes	Yes	Yes	8
Jeong UC et al. 2015 [21]	No	Yes	No	Yes	No	No	No	Yes	Yes	Yes	Yes	6
Kendall KD et al. 2014 [22]	Yes	Yes	Yes	Yes	No	No	No	Yes	Yes	Yes	Yes	8
Kim B and Yim 2020 [23]	Yes	Yes	Yes	Yes	Yes	No	Yes	Yes	No	Yes	Yes	9
Lee SW et al. 2014 [24]	Yes	Yes	No	Yes	No	No	No	Yes	No	Yes	Yes	6

PEDro questionnaire items → 1: eligibility criteria; 2: random assignment; 3: hidden allocation; 4: baseline comparison; 5: blind subjects; 6: blind therapist; 7: blind evaluators; 8: adequate follow-up; 9: intention-to-treat analysis; 10: comparison between groups; 11: point estimates and variability.

**Table 3:** Summary of studies included in the systematic review, participants, and intervention characteristics.

First Author, Year, and Country of Publication	Study Design	Participants (Baseline Sample Side and Characteristics)	Intervention	Outcomes	Results (Pre vs. Post)
Bade M et al. 2017, USA [18]	Random controlled trial	n_i_ = 90 (37♀ and 53♂); NSLBP ≥ 2 in NPRS and disability ≥ 20% in ODI CG: n_i_ = 43 (16♀ and 27♂, 11 dropout → n_f_ = 32) Age (mean ± SD): 48.1 ± 2.4 y Height (mean ± SD): 1.7 ± 0.0 m Weight (mean ± SD): 78.5 ± 3.1 Kg Symptom duration (media ± SD): 19.7 ± 7.2 Wk IG: n_i_ = 47 (21♀ and 26♂, 7 dropout → n_f_ = 40) Age (mean ± SD): 44.8 ± 2.3 y Height (mean ± SD): 1.7 ± 0.0 m Weight (mean ± SD): 81.3 ± 4.7 Kg Symptom duration (media ± SD): 20.3 ± 6.5 Wk	CG: MT, coordination, strengthening and resistance trunk ex., PNS mobilizations, tractions, aerobic ex., flexion ex., fitness, centralization and directional preference ex. and procedures	Pain: NPRS Disability: ODI GROC PASS	CG: changes from baseline ↓ NPRS (mean ± SD): 5.4 ± 0.3 vs. 1.9 ± 1.6 ↓ ODI (mean ± SD): 36.7 ± 2.1 vs. 11.9 ± 7.1
			GI: CG intervention + HM strengthening + hip MT (mobilization degree III-IV, 30 s/technique; A-P mobilization with traction, traction and mobilization P-A in PP)		IG: changes from baseline ↓ NPRS (mean ± SD): 5.1 ± 0.3 vs. 1.1 ± 1.1 ↓ ODI (mean ± SD): 36.4 ± 1.5 vs. 9.1 ± 8.5 IG vs. CG ↓* NPRS (mean ± SD): 1.1 ± 1.1 vs. 1.9 ± 1.6 ↓* ODI (mean ± SD): 9.1 ± 8.5 vs. 11.9 ± 7.1 ↓* GROC (medium (1st quartile, 3rd quartile)): 6.0 (5.0, 7.0) vs. 5.0 (4.9, 6.0) ↔ PASS: yes (36 vs. 26), no (3 vs. 1), missing (6 vs. 12)
Cai C et al. 2017, Singapore [19]	Random controlled trial, simple blind	n_i_ = 84 (42♀ and 42♂) NSCLBP CG: -LE: n_i_ = 28 (4 dropout → n_f_ = 24) Age (mean ± SD): 26.1 ± 4.1 y Weight (mean ± SD): 61.7 ± 10.8 Kg BMI (mean ± SD): 21.8 ± 2.4 Kg/m^2^ -LS: n_i_ = 28 (3 dropout → n_f_ = 25) Age (mean ± SD): 26.9 ± 6.4 y Weight (mean ± SD): 60.3 ± 12.1 Kg BMI (mean ± SD): 21.9 ± 2.4 Kg/m^2^ IG: n_i_ = 28 (3 dropout → n_f_ = 25) Age (mean ± SD): 28.9 ± 5.3 y Weight (mean ± SD): 61.7 ± 12.6 Kg BMI (mean ± SD): 21.7 ± 2.4 Kg/m^2^	CG: *-LE*: Lumbar extensor strengthening ex. *-LS:* lumbopelvic motor control ex.	Pain: NPRS Disability: PSFS LL strength: dynamometry LE resistance: EMG Activation of trunk-stabilizing muscles: US	CG (LE and LS): changes from baseline ↓* NPRS (mean ± SD): -LE: 3.44 ± 0.87 vs. 0.76 ± 0.78 -LS: 3.62 ± 1.13 vs. 0.65 ± 0.56 ↑* PSFS (mean ± SD): -LE: 6.71 ± 0.92 vs. 8.65 ± 0.85 -LS: 6.62 ± 0.90 vs. 8.81 ± 0.80 ↑ LL strength ↑* LE resistance ↑* Activation of trunk-stabilizing muscles IG: changes from baseline ↓* NPRS (mean ± SD): 3.48 ± 1.00 vs. 0.32 ± 0.48 ↑* PSFS (mean ± SD): 6.52 ± 0.90 vs. 9.23 ± 0.65 ↑* LL strength ↑* LE resistance ↑* Activation of trunk-stabilizing muscles IG vs. CG (LE and LS) ↓* NPRS (mean ± SD): 0.32 ± 0.48 vs. 0.76 ± 0.78 and 0.65 ± 0.56 ↑* PSFS (mean ± SD): 9.23 ± 0.65 vs. 8.65 ± 0.85 and 8.81 ± 0.80 ↑ LL strength ↑* LE endurance ↔ Activation of trunk-stabilizing muscles
			IG: HM and knee strengthening ex.		
Fukuda TY et al. 2021, Brazil [20]	Random controlled trial, simple blind	n_i_ = 70 (37♀ and 33♂) NSCLBP CG: n_i_ = 35 (3 dropout → n_f_ = 32) Age (mean ± SD): 35.2 ± 12.5 y Height (mean ± SD): 1.6 ± 0.1 m Weight (mean ± SD): 72.6 ± 15.6 Kg BMI (mean ± SD): 25.3 ± 4.6 Kg/m^2^ Symptom duration (mean ± SD): 6.9 ± 8.1 month IG: n_i_ = 35 (4 dropout → n_f_ = 31) Age (mean ± SD): 40.2 ± 12.4 y Height (mean ± SD): 1.7 ± 0.1 m Weight (mean ± SD): 75.8 ± 15.9 Kg BMI (mean ± SD): 25.9 ± 5.4 Kg/m^2^ Symptom duration (mean ± SD): 8.1 ± 8.9 month	CG: MT (P-A-C mobilization degree III of L1-L5, 5 reps/1 min following Maitland method and myofascial liberation) Segmentary lumbar stabilization ex.	Pain: VAS Disability: RMDQ HM strength: dynamometry Kinematic analysis of gait (LL, trunk, and pelvis)	CG: changes from baseline ↓ VAS (mean ± SD): 5.6 ± 2.1 vs. 2.9 ± 2.0 ↓ RMDQ (mean ± SD): 9.1 ± 4.7 vs. 4.3 ± 3.5 ↑ HM strength ↔ Kinematic analysis IG: changes from baseline ↓ VAS (mean ± SD): 5.5 ± 2.1 vs. 2.3 ± 2.2 ↓ RMDQ (mean ± SD): 8.5 ± 4.6 vs. 4.5 ± 4.4 ↑ HM strength ↔ Kinematic analysis IG vs. CG ↔ VAS (mean ± SD): 2.3 ± 2.2 vs. 2.9 ± 2.0 ↔ RMDQ (mean ± SD): 4.5 ± 4.4 vs. 4.3 ± 3.5 ↔ HM strength ↔ Kinematic analysis
			IG: CG intervention + HM strengthening ex.		
Jeong UC et al. 2015, Korea [21]	Random controlled trial	n_i_ = 40♀ NSLBP ≥ 5 in VAS and disability ≥ 20% in ODI CG: n_i_ = 20♀ (0 dropout → n_f_ = 20) Age (mean ± SD): 41.2 ± 6.7 y Height (mean ± SD): 159.9 ± 4.7 cm Weight (mean ± SD): 56.6 ± 4.2 Kg IG: n_i_ = 20♀ (0 dropout → n_f_ = 20) Age (mean ± SD): 41.2 ± 5.5 y Height (mean ± SD): 161.5 ± 6.0 cm Weight (mean ± SD): 59.7 ± 7.2 Kg	CG: Lumbar stabilization ex. (2 sets/20 reps/10 s)	Disability: ODI Lumbar strength: M3 Balance: Tetrax	CG: changes from baseline ↓ ODI (mean ± SD) (pre–post value): 4.5 ± 2.4 ↑ Lumbar strength ↑ Balance IG: changes from baseline ↓ ODI (mean ± SD) (pre–post value): 9.9 ± 3.2 ↑Lumbar strength ↑ Balance IG vs. CG ↓* ODI (mean ± SD) (pre–post value): 9.9 ± 3.2 vs. 4.5 ± 2.4 ↑* Lumbar strength ↑* Balance
			IG: CG intervention + HM strengthening ex.		
Kendall KD et al. 2014, Canada [22]	Random controlled trial	n_i_ = 80 (42♀ and 38♂); NSCLBP ≥ 5 in VAS CG: n_i_ = 40 (18♀ and 22♂, 4 dropout → n_f_ = 36) Age (95%CI): 33 (33, 41) y Height (95%CI): 172 (169, 175) cm Weight (95%CI): 73 (68, 78) Kg Symptom duration (95%CI): 4 (3, 6) y IG: n_i_ = 40 (24♀ and 16♂, 5 dropout → n_f_ = 35) Age (95%CI): 41 (37, 45) y Height (95%CI): 170 (167, 173) cm Weight (95%CI): 77 (71, 83) Kg Symptom duration (95%CI): 7 (4, 10) y	CG: Lumbopelvic motor control (transverse, multifidus and pelvic floor coordination)	Pain: VAS Disability: ODI HM strength: dynamometry Trendelenburg Test	CG: changes from baseline ↓* VAS (mean (95%CI)): 57 (54, 61) vs. 37 (31, 41) mm ↓* ODI (mean (95%CI)): 22 (19, 24) vs. 14 (11, 17) ↔ HM strength ↔ Trendelenburg Test IG: changes from baseline ↓* VAS (mean (95%CI)): 55 (51, 58) vs. 30 (24, 36) mm ↓* ODI (mean (95%CI)): 20 (17, 23) vs. 12 (10, 14) ↑* HM strength ↔ Trendelenburg Test IG vs. CG ↔ VAS (mean (95%CI)): 30 (24, 36) vs. 37 (31, 41) mm ↔ ODI (mean (95%CI)): 12 (10, 14) vs. 14 (11, 17) ↑* HM strength ↔ Trendelenburg test
			IG: CG intervention + HM strengthening ex.		
Kim B and Yim 2020, Korea [23]	Randomized controlled trial, doble blind	n_i_ = 75 (32♀ and 34♂); NSCLBP ≥ 3 in VAS CG: n_i_ = 25 (5 dropout → n_f_ = 20) Age (mean ± SD): 47.7 ± 8.5 y Height (mean ± SD): 167.7 ± 8.1 cm Weight (mean ± SD): 67.6 ± 8.7 Kg BMI (media ± SD): 23.9 ± 1.0 Kg/m^2^ IG: *-*SIG: n_i_ = 25 (3 dropout → n_f_ = 22) Age (mean ± SD): 47.0 ± 9.4 y Height (mean ± SD): 166.5 ± 2.1 cm Weight (mean ± SD): 66.0 ± 9.2 Kg BMI (mean ± SD): 23.6 ± 1.5 Kg/m^2^ -FIG: n_i_ = 25 (1 dropout → n_f_ = 24) Age (mean ± SD): 47.5 ± 9.7 y Height (mean ± SD): 164.7 ± 8.2 cm Weight (mean ± SD): 65.4 ± 10.4 Kg BMI (mean ± SD): 23.9 ± 1.6 Kg/m^2^	CG: Core stability ex. (30 min, 3 session/sem, 6 sem, 10reps/7–8sec) Placebo (light palpation of the lumbosacral region)	Pain: VAS Disability: ODI and RMDQ HM flexibility: TTT, MTT, OT, and FAIRT Balance: OLST QoL: SF-36	CG: changes from baseline ↓* VAS (mean ± SD): 5.85 ± 1.16 vs. 2.92 ± 0.61 ↓* ODI (mean ± SD): 58.20 ± 5.27 vs. 36.70 ±5.12 ↓* RMDQ (mean ± SD): 11.40 ± 2.28 vs. 5.55 ± 1.82 ↑* HM flexibility ↑* OLST ↑* SF-36 SIG and FIG: changes from baseline ↓* VAS SIG (mean ± SD): 6.12 ± 1.02 vs. 2.37 ± 0.69 FIG (mean ± SD): 5.95 ± 1.09 vs. 2.37 ± 0.67 ↓* ODI SIG (mean ± SD): 56.91 ± 6.92 vs. 30.18 ± 7.66 FIG (mean ± SD): 57.67 ± 6.50 vs. 29.25 ± 7.66 ↓* RMDQ SIG (mean ± SD): 11.23 ± 2.62 vs. 3.54 ± 1.59 FIG (mean ± SD): 11.29 ± 1.85 vs. 3.58 ± 1.35 ↑* HM flexibility ↑* OLST ↑* SF-36 SIG vs. CG ↓* VAS (mean ± SD): 2.37 ± 0.69 vs. 2.92 ± 0.61 ↓* ODI (mean ± SD): 30.18 ± 7.66 vs. 36.70 ± 5.12 ↓* RMDQ (mean ± SD): 3.54 ± 1.59 vs. 5.55 ± 1.82 ↔ HM flexibility ↑* OLST ↑* SF-36 FIG vs. CG ↓* VAS (mean ± SD): 2.37 ± 0.67 vs. 2.92 ± 0.61 ↓* ODI (mean ± SD): 29.25 ± 7.66 vs. 36.70 ± 5.12 ↓* RMDQ (mean ± SD): 3.58 ± 1.35 vs. 5.55 ± 1.82 ↑* HM flexibility ↑* OLST ↑* SF-36 FIG vs. SIG ↔VAS (mean ± SD): 2.37 ± 0.67 vs. 2.37 ± 0.69 ↔ ODI (mean ± SD): 29.25 ± 7.66 vs. 30.18 ± 7.66 ↔ RMDQ (mean ± SD): 3.58 ± 1.35 vs. 3.54 ± 1.59 ↑* HM flexibility ↔ OLST ↔ SF-36
			IG: *-SIG:* core stability ex. + HM strengthening ex. *FIG:* core stability ex. + HM static stretching ex.		
Lee SW et al. 2014, Korea [24]	Randomized controlled trial	n_i_ = 78 CLBP CG: n_i_ = 31 (6 dropout → n_f_ = 25) -CG_LS_*:* n_i_ = 20 (4 dropout → n_f_ = 16) Age (mean ± SD): 50.0 ± 11.4 y Height (mean ± SD): 161.9 ± 7.7 cm Weight (mean ± SD): 60.9 ± 9.8 Kg BMI (mean ± SD): 23.2 ± 2.8 Kg/m^2^ -CG_IN_*:* n_i_ = 11 (2 dropout → n_f_ = 9) Age (mean ± SD): 59.3 ± 17.3 y Height (mean ± SD): 161.0 ± 8.3 cm Weight (mean ± SD): 59.5 ± 10.0 Kg BMI (mean ± SD): 22.8 ± 2.9 Kg/m^2^ IG: n_i_ = 47 (3 dropout → n_f_ = 44) -IG_LS_*:* n_i_ = 25 (2 dropout → n_f_ = 23) Age (mean ± SD): 54.9 ± 10.6 y Height (mean ± SD): 161.0 ± 7.1 cm Weight (mean ± SD): 61.9 ± 9.8 Kg BMI (mean ± SD): 23.8 ± 2.8 Kg/m^2^ -IG_IN_: n_i_ = 22 (1 dropout → n_f_ = 21) Age (mean ± SD): 61.0 ± 13.2 y Height (mean ± SD): 159.7 ± 6.0 cm Weight (mean ± SD): 59.4 ± 8.9 Kg BMI (mean ± SD): 23.3 ± 2.6 Kg/m^2^	CG: Lumbar stability ex. (4 ex./4 sets/4 reps/ 10 s, 30 s rest)	Pain: VAS Disability: modified ODI	CG: changes from baseline ↓* VAS CG_LS_ (mean ± SD): 55.30 ± 10.70 vs. 45.6 ± 10.30 CG_IN_ (mean ± SD): 61.00 ± 10.00 vs. 27.60 ± 9.80 ↓* ODI CG_LS_ (mean ± SD): 25.60 ± 12.30 vs. 21.70 ± 10.70 CG_IN_ (mean ± SD): 30.60 ± 18.80 vs. 18.30 ± 11.10 IG: changes from baseline ↓* VAS IG_LS_ (mean ± SD): 55.70 ± 8.90 vs. 39.60 ± 7.50 IG_IN_ (mean ± SD): 58.90 ± 8.60 vs. 43.3 ± 12.00 ↓* ODI IG_LS_ (mean ± SD): 23.80 ± 10.50 vs. 17.50 ± 8.10 IG_IN_ (mean ± SD): 25.9 ± 15.80 vs. 19.80 ± 12.10 IG vs. CG ↓ VAS ↓ ODI
			IG: CG intervention + HM strengthening ex. + hip mobility ex.		

Abbreviations: ↓: decrease; ↑: increase; ↔: without change; *: statistically significant change (p < 0,05); n_i_: initial sample side; n_f_: final sample side; ♀: women; ♂: men; CG: control group; IG: intervention group; SD: Standard Deviation; m: meters; Kg: kilograms; Wk: week; y: year; cm: centimeter; BMI: body mass index; NSLBP: non-specific low back pain; NSCLBP: non-specific chronic low back pain; CLBP: chronic low back pain; NPRS: Numeric Pain Rating Scale; ODI: Oswestry Disability Index; MT: manual therapy; ex.: exercise; PNS: peripheral nervous system; HM: hip muscles; A-P: antero-posterior; P-A: postero-anterior; PP: prone position; GROC: Global Rating of Change; PASS: patient acceptable symptom state; LE: lumbar extensor; LS: lumbar stability; PSFS: patient-specific functional scale; LL: lower limb; EMG: electromyography; US: ultrasound; P-A-C: postero-anterior-central; L1: first lumbar vertebra; L5: fifth lumbar vertebra; reps: repetitions; min: minutes; RMDQ: Roland–Morris Disability Questionnaire; VAS: Visual Analogue Scale; s: second; CI: confidence interval; SIG: strength intervention group; FIG: flexibility intervention group; TTT: toe touch test; MTT: Modified Thomas Test; OT: Ober test; FAIRT: Flexion adduction internal rotation test; OLST: one-leg standing test; QoL: quality of life; IN: lumbar instability.

**Table 4:** Characteristics of hip muscle strengthening interventions.

First Author, Year and Country of Publication	Exercise	Volume and Intensity	Frequency (Days/Week)	Time (Minutes/Session)	Duration (Weeks)	Supervision
Bade M et al. 2017, USA [18]	Clam in side lying with ER Quadruped hip extension Unilateral bridge Home ex.	2 sets of 12–15 reps	7 -Home ex. twice a day	-	-	Yes -Home ex. with instructions
Cai C et al. 2017, Singapore [19]	Device for strengthening hip abd, extensor, and knee extensor Home ex.: -single-leg squat -wall sit	Supervised: 3 sets of 10 reps, 2 min rest 10 RM Home ex.: 3 sets of 10 rep, 2.5 Kg single-leg squat, and 5 Kg wall sit	Supervised: 2 Home ex.: 5	45	8	Yes -Home ex. with instructions
Fukuda et al. 2021, Brazil [20]	Clam in side lying with ER Lateral straight leg rise with ankle weight Squat with ER Monster Walk with ER	3 sets of 10 reps 70% RM Ex. with ER: maximum resistance that enables 10 reps	2	45	5	Yes
Jeong UC et al. 2015, Korea [21]	Gluteus maximus and gluteus medius ex. 3 Wk without resistance and 3 Wk with resistance	2 sets of 15 reps	3	50	6	Yes
Kendal KD et al. 2014, Canada [22]	Controlled with US (not specified) Home ex.: open and close kinetic chain hip ex.	Not specified	Supervised: 1 Home ex.: not specified	Not specified	6	Yes -Home ex. with instructions
Kim B and Yim 2020, Korea [23]	FIG: HM static stretching (hamstring, iliopsoas, piriformis, and tensor fasciae latae) SIG: HM strengthening ex. (side lying hip abd with IR, prone heel squeeze, quadruped hip extension, standing gluteal squeeze)	3 reps of 30 s 10 s rest	3	45	6	Yes
Lee SW et al. 2014, Korea [24]	To increase ROM: 4 open kinetic chain hip ex. 6 strengthening ex. with ER	3 sets of 10 reps, 1 min rest 75% RM	3	ROM ex.: 20 Strengthening ex.: not specified	6	Yes

Abbreviations: ER: elastic resistance; Reps: repetitions; ex.: exercise; abd: abduction, add: adduction; min: minutes; s: seconds; RM: maximal repetition; Kg: kilograms; Wk: week; US: ultrasound; FIG: flexibility intervention group; SIF: strength intervention group; HM: hip muscle; ROM: range of movement.

**Table 5:** Hip muscle strengthening intervention protocol in patients with LBP.

	Warm-Up	Central Part	Return to Calm
Exercises	Joint mobility Muscular activation	HM strengthening: Squat Monster Walk Quadruped hip extension Clam in side lying Bridge	Relax Static stretch Manual therapy
Intensity	Minimum	75–80% RM	
Volume		2–3 sets/8–12 reps for ex. 1 min rest	
Time	5–10 min	45–50 min	5–10 min
Frequency	3–4 days/week, with 1–2 days of rest between sessions		
Observations	The volume and intensity should be increased as the patient improves, increasing the number of repetitions and/or loads (elastic resistance or weight)		
Abbreviations	RM: maximal repetition; reps: repetitions		

## Data Availability

Not applicable.
